# Morphometric analyses reveal synonymy of two monotypic genera, *Huangiella* and *Tumoris* (Acari, Eriophyoidea, Eriophyidae)

**DOI:** 10.3897/zookeys.102.948

**Published:** 2011-06-02

**Authors:** Chin-Fah Wang, Chi-Chien Kuo, Ming-Luen Jeng, Kun-Wei Huang

**Affiliations:** 1 General Education Center, National Chiayi University, Chiayi 600, Taiwan, R.O.C.; 2 Department of Zoology, National Museum of Natural Science, Taichung 404, Taiwan, R.O.C.

**Keywords:** Multivariate analysis, Eriophyid mites, Lanyu, Green Island, valid name, *Tumoris sanasaii*

## Abstract

Morphological variation of *Huangiella lanyuensis* (Huang, 2001) and *Tumoris sanasaii* Huang, 2001 from Taiwan was analyzed using multivariate statistical methods. We show that these species are the same and propose to use the name *Tumoris sanasaii*. No significant differences between populations from Lanyu and Green Island (type localities for *Huangiella lanyuensis* and *Tumoris sanasaii*, respectively) were found; however, mites from Yangmingshan (northern Taiwan) differed substantially from these two groups. Synonymy resulted from our study is as follows: *Huangiella* Kammerer, 2006 is a junior synonym of *Tumoris* Huang, 2001; *Absentia lanyuensis* Huang, 2001 is a junior synonym of *Tumoris sanasaii* Huang, 2001. We also study the sexual variation of populations from Green Island. The result showed the females significantly larger than the males at 17 variables.

## Introduction

Eriophyid mites, also known as gall, blister, erineum, bud and rust mites, have more than 200 genera and about 3700 described species worldwide (De Lillo and Amrine 2003). They differ from the other mites by having only two pairs of legs and by their entirely herbivorous habits. The body is minute in size (80–250 µm) with most of the body structures reduced. These characteristics make them a difficult taxon to study and the actual diversity may be several folds higher than currently known (Amirne 1996).

Huang (2001a, b) established two monotypic eriophyid genera *Absentia* and *Tumoris* based on *Absentia lanyuensis* Huang and *Tumoris sanasaii* Huang, respectively. The former species was reported from *Symplocos cochinchinensis philippinensis* (originally misidentified as *Symplocos cochinchinensis cochinchinensis*) in Lanyu (Orchid Island) (Huang 2001a), whereas the latter was collected from Green Island (Ludao) from the same plant subspecies (Huang 2001b). No subsequent species has been added to these two genera since then. The name *Absentia* was later found preoccupied and a replacement name, *Huangiella*,was proposed by Kammerer (2006).

Here we add another mite population from *Symplocos cochinchinensis cochinchinensis* in northern Taiwan and made several morphometric analyses to determine if these groups are distinct. Sexual variation in the Green Island population was also analyed by multivariate analysis to reveal the morphological difference between sexes.

## Materials and methods

### Acquisition of specimens and preparation of slide specimens

Specimens used in the present study were collected from Lanyu (22°2'45", 121°31'50") in 31-Aug.-1994, 18-Aug.-1998 and 28-May-2008, from Green Island (22°39'52", 121°29'17") in 5-Jun.-2000 (collected from different trees), and from Yangmingshan (25°10'15", 121°34'26") in 18-Aug.-1999 and 24-Aug.-1999 (prepared and measured by CFW and KWH). Specimen mounting was followed by Huang (2008). Every specimen was mounted dorso-ventrally on a single slide.

Through microscopic examination, 136 out of the 246 slides prepared from the mite samples collected from *Symplocos cochinchinensis philippinensis* on Lanyu and Green Island were found to be the species in question. Eighty-five individuals allowing measurements of all morphometric variables, including 32 females from Lanyu (LF), 22 males and 31 females from Green Island (GM and GF, respectively), were chosen for morphometric measurement and analysis. We also prepared mite specimens collected from *Symplocos c. cochinchinensis* in Yangmingshan (north Taiwan). Out of 24 individuals, 16 females (YF) were chosen for measurements.

### Variable Selection and Measurement

**Figure 1.**
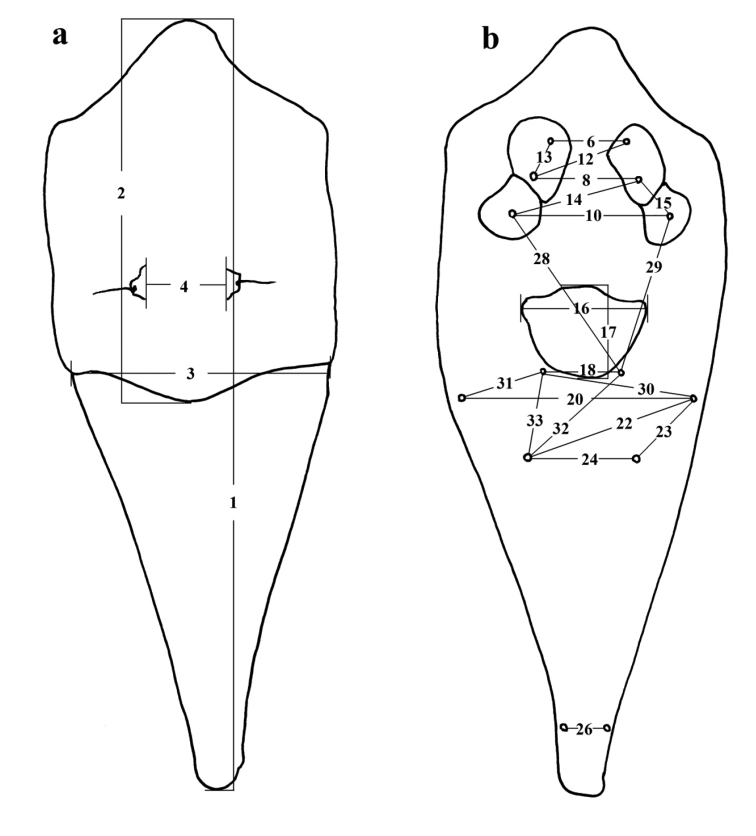
Contour drawing of *Tumoris sanasaii* Huang, 2001 and the measurement of the 33 variables used in this study. The number corresponds to the number of the variable listed in Table 1. **a** dorsal view **b** ventral view.

Thirty-three variables for morphometric analyses were selected and measured (Table 1). The variables includes ones based on the the homologous landmarks or length of setae commonly used in taxonomic descriptions. The distance between setal tubercles was measured by truss method (Strauss and Bookstein 1982; Huang et al. 1996) (Fig. 1), and was doubly measured in opposite orientations then averaged. All morphometric data in this study were shown in micrometers (µm).

**Table 1.** 33 morphometric characters and their abbreviation used in this study.

*Variables*		*Abbreviation*
1	body length	BL
2	shield length	SL
3	shield width	SW
4	distance between the dorsal tubercles	Dt-Dt
5	dorsal setae length	Ds.l
6	distance between the 1st coxal tubercles	Ct1-Ct1
7	1st coxal setae length	Ct1.l
8	distance between the 2nd coxal tubercles	Ct2-Ct2
9	the 2nd coxal setae length	Ct2.l
10	distance between the 3rd coxal tubercles	Ct3-Ct3
11	the 3rd coxal setae length	Ct3.l
12	cross distance from the 1st to the 2nd coxal tubercles	Ct1\Ct2
13	distance from the 1st to the 2nd coxal tubercles	Ct1-Ct2
14	cross distance from the 2nd to the 3rd coxal tubercles	Ct2\Ct3
15	distance from the 2nd to the 3rd coxal tubercles	Ct2-Ct3
16	genital width	Gs.W
17	genital length	Gs.L
18	distance between the genital tubercles	Gt-Gt
19	genital setae length	Gs.l
20	distance between the lateral tubercles	Lt-Lt
21	lateral setae length	Lt.l
22	cross distance from the lateral tubercles to the 1st ventral tubercles	Lt\Vt1
23	distance from the lateral tubercles to the 1st ventral tubercles	Lt-Vt1
24	distance between the 1st ventral tubercles	Vt1-Vt1
25	the 1st ventral setae length	Vt1.l
26	distance between the 3rd ventral tubercles	Vt3-Vt3
27	the 3rd ventral setae length	Vt3.l
28	cross distance from the 3rd coxal tubercles to the genital tubercles	Ct3\Gt
29	distance from the 3rd coxal tubercles to the genital tubercles	Ct3-Gt
30	cross distance from the genital tubercles to the lateral tubercles	Gt\Lt
31	distance from the genital tubercles to the lateral tubercles	Gt-Lt
32	cross distance from the genital tubercles to the 1st ventral tubercles	Gt\Vt1
33	distance from the genital tubercles to the 1st ventral tubercles	Gt-Vt1

### Analysis

We evaluated geographic and sexual variations in morphology with multivariate analysis of variance (MANOVA). Morphometric data obtained from 101 mites from three localities was analyzed. Females of Yangmingshan, females of Lanyu, and females of Green Island (YF+LF+GF) were used to test if they are the same species, whereas the individuals from Green Island (GM and GF) were used to detect the sexual variation. Morphometric measurements (including distance between setal bases and the lengths of setae) were standardized by subtracted the mean. Principal components analysis (PCA) was then applied to reduce multicollinearity. Variation among populations in derived orthogonal principal components was firstly identified with MANOVA. Once a significant result was detected, pair-wise MANOVA tests after Bonferroni adjustment (*α*-level: 0.05 divided by *n* comparisons) were followed to identify the pair(s) leading to the difference. We also created a canonical centroid plot, which provides a convenient way for simultaneously inspect differences among populations (the canonical centroid plot depicted the 95% confidence interval for centroid of each population and an overlap of boundary represents no difference in response variables).

We then applied analysis of variance (ANOVA) to determine which response variable (i.e. PC1, PC2, etc.) accounted for the variation. Lastly, differences in those morphometric measurements with high absolute loadings in selected principal components (those that significantly differed among populations) were tested with ANOVA or *t*-test. For the MANOVA test, normality of response variables (PC values for morphometric measurements) was confirmed with Shapiro–Wilk test, and multivariate outliers were identified with jackknifed Mahalanobis distance. All the procedures were implemented in JMP 8.0 (SAS Institute Inc., Cary, N.C.).

## Results and discussion

### Geographical variation

**Figure 2.**
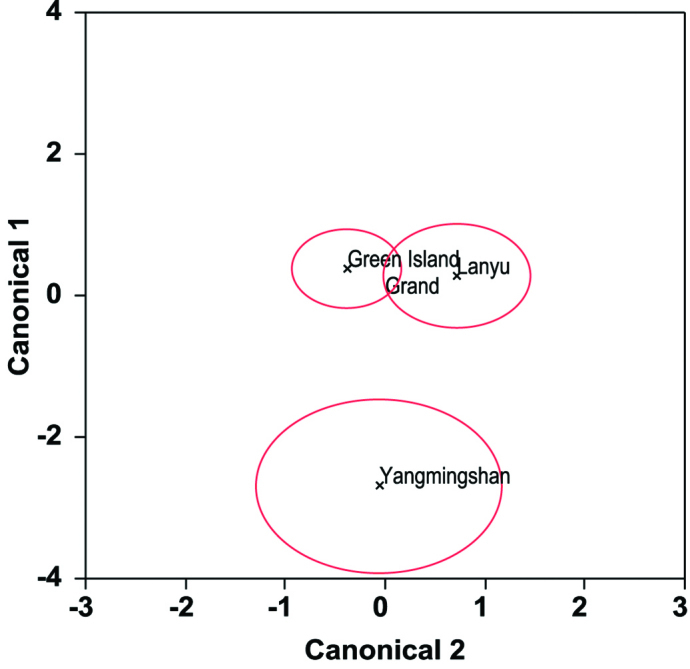
Canonical centroid plot for geographical variation in morphometric characteristics for Yangmingshan (YF), Lanyu (LF) and Green Island (GF).

We applied PCA to reduce the dimensionality in 33 standardize morphometric variables. The three principal axes (PC1–3) were normally distributed within the three locations (Shapiro–Wilk test, W ranged from 0.92 to 0.97, all *P*> 0.05), and there were no outliers in PC values. PC1, PC2, and PC3 varied significantly among the three locations (MANOVA Wilks’ λ test: *F*6,60= 6.04, *P*<0.0001). Canonical centroid plot revealed that morphological characteristics in Yangmingshan (YF) statistically differed from those in Lanyu (LF) and Green Island (GF) (pair-wise MANOVA: *F*3,11= 13.54 and *F*3,20= 14.56, respectively; both *P*< 0.001), whereas the latter two cannot be distinguished from each other (*F*3,27= 2.59, *P*> 0.05) (Fig. 2). Further ANOVA showed that these variations were due to the differences in PC1 (*F*2,32= 8.83, *P*< 0.001) and PC3 (*F*2,32= 8.13, *P*< 0.005), but cannot be explained by PC2 (*F*2,32= 0.43, *P*= 0.65). Absolute values of loadings were higher in Bl, Lt-Lt, Lt\Vt1, Vt1-Vt1, Gt\Lt, and Gt-Lt for PC1, and higher in Sw and Ct1-Ct2 for PC3 (Table 2). Among these variables (using original measurements), Bl (YF: 151.7±3.1 (mean±1SD), 147.9–155.2 (range); LF+GF: 135.3±13.2, 114.3–178.1; *t*-test, *t*= 2.44, *P*< 0.05) and Sw (YF: 61.5±2.0, 58.7–63.3; LF+GF: 51.7±5.6, 39.5–66.8; *t*= 3.45, *P*< 0.005) in YF were significantly different (all were larger) from those in LF and GF (combined due to similarity in morphology).

### Sexual variation

The three principal axes derived from 33 standardized morphometric variables were normally distributed (W ranged from 0.93 to 0.98, all *P*> 0.05), and varied significantly between the males (GM) and the females (GF) in Green Island (MANOVA *F*3,34= 46.51, *P*<0.0001) (Fig. 3). Sexual differences were observed in PC1 (*t*-test, *t*= 11.87, *P*< 0.001), but not in PC2 (*t*= -0.51, *P*= 0.62) and PC3 (*t*= 0.14, *P*= 0.89). Absolute loadings were higher in Bl, Sl, Ds.l, Ct3-Ct3, Ct1\Ct2, Gs.w, Gs.l, Gt-Gt, Lt-Lt, Lt\Vt1, Lt-Vt1, Vt1-Vt1, Ct3\Gt, Ct3-Gt, Gt\Lt, Gt-Lt, and Gt\Vt1 for PC1 (Table 2). Sexual variation was observed in all these 17 variables (*t*-test), with the females significantly larger than the males (Table 3).

**Figure 3.** Canonical centroid plot for sexual variation in morphometric characteristics for male (M) and female (F) of Green Island.

### Identity of the geographical groups

The analyses on geographical groups revealed no significant differences between the Lanyu and Green Island populations, indicating that *Huangiella lanyuensis* and *Tumoris sanasaii* are actually the same. On the other hand, the group from Yangmingshan, previously identified by KWH as *Tumoris sanasaii*, is distinct. Though sharing similar diagnostic characters with *Tumoris sanasaii*, the Yangmingshan group differs significantly from the true *Tumoris sanasaii* by morphometry. The former group feeds on a different subspecies of host plant in the temperate northern Taiwan, in contrast to true *Tumoris sanasaii* living on subtropical or tropical Green Island and Lanyu. At present it is difficult to determine whether it is intra- or inter-specific difference. We would suggest their differentiation being above subspecies level because eriophyid mites have more rapid evolution rate than their host plants. A further study using multidisciplinary approaches would be required to solve the problem.

Owing to the reduced morphological structures and minute size of eriophyids, descriptive diagnosis is usually unsatisfactory in differentiating closely related species. Molecular identification is also difficult owing to the hardness to isolate a single individual of an identified species from a mite community without making a slide. Morphometric analyses thus provide a reasonable option with balance in effectiveness and efficiency. The present and many previous studies have proved morphometrics a useful tool in eriophyoid classification (Huang et al. 1996; Magud et al. 2007; Skoracka et al. 2002; Navia et al. 2006, 2009; Skoracka 2009a, b).

**Table 2.** Loadings in principal components of geographical and sexual variations in morphometric variables of Eriophyoid mites in Taiwan (only PCs that significantly differ among populations were shown).

Morphometric variables	Geographical variation		Sexual variation
	PC1	PC3	PC1
Bl	-0.85	0.27	0.60
Sl	0.30	0.51	0.80
Sw	-0.02	-0.64	0.59
Dt-Dt	-0.33	0.47	0.23
Ds.l	-0.26	0.55	0.61
Ct1-Ct1	0.35	0.18	0.49
Ct1.l	-0.01	0.19	0.27
Ct2-Ct2	0.37	-0.12	0.55
Ct2.l	0.49	-0.21	0.41
Ct3-Ct3	0.23	-0.19	0.66
Ct3.l	0.32	-0.35	0.20
Ct1\Ct2	0.54	0.27	0.60
Ct1-Ct2	-0.15	0.61	0.41
Ct2\Ct3	0.40	-0.12	0.58
Ct2-Ct3	-0.07	-0.05	0.40
Gs.w	0.57	0.03	0.90
Gs.l	0.36	0.02	0.90
Gt-Gt	-0.05	-0.10	0.74
Gs.l 2	-0.47	-0.21	0.36
Lt-Lt	0.66	0.48	0.83
Lt.l	0.02	-0.49	0.20
Lt\Vt1	0.71	0.18	0.85
Lt-Vt1	0.33	-0.19	0.65
Vt1-Vt1	0.60	0.27	0.67
Vt1.l	-0.53	-0.11	0.26
Vt3-Vt3	0.04	0.15	0.14
Vt3.l	0.37	-0.31	-0.05
Ct3\Gt	0.51	-0.49	0.88
Ct3-Gt	0.39	-0.40	0.72
Gt\Lt	0.69	0.34	0.89
Gt-Lt	0.70	0.50	0.69
Gt\Vt1	0.60	-0.17	0.85
Gt-Vt1	0.40	-0.24	0.37

**Table 3.** The 17 characters with significant difference between male and female of *Tumoris sanasaii* in Green Island.

Morphometricvariables	Male		Female		t-value
	mean±1SD	range	mean±1SD	range	
Bl	126.6±6.2	114–137	132.7±9.4	114–159	2.32*
Sl	50.0±2.7	45–54	54.7±3.1	50–60	5.05***
Ds.l	6.6±1.1	4.9–8.7	8.2±1.8	4.9–12	3.36**
Ct3-Ct3	16.8±0.8	14–18	18.5±1.2	16–20	4.96***
Ct1\Ct2	9.4±0.6	8.3–11	10.2±1.0	8.7–13	2.82*
Gs.w	14.0±1.6	11–18	19.0±1.3	16–21	10.78***
Gs.l	4.7±1.5	2.6–7.9	12.3±1.0	8.9–16	13.36***
Gt-Gt	11.1±1.4	9–15	14.0±1.1	12–16	6.95***
Lt-Lt	34.0±1.4	31–37	37.8±2.2	33–41	6.39***
Lt\Vt1	29.4±1.1	26–31	33.7±2.3	27–38	7.26***
Lt-Vt1	18.6±1.5	16–21	20.8±2.2	14–23	3.48**
Vt1-Vt1	15.4±1.2	12–17	18.7±2.1	16–24	5.92***
Ct3\Gt	21.0±1.0	19–23	24.8±1.2	23–28	10.91***
Ct3-Gt	15.8±1.3	13–18	18.7±1.8	17–25	5.68***
Gt\Lt	22.3±1.1	19–24	25.9±1.2	24–29	9.31***
Gt-Lt	10.7±1.0	9–12	12.3±1.2	10–14	4.49***
Gt\Vt1	19.5±1.1	18–21	22.1±1.3	20–25	6.80***

*P<0.05; **P<0.005; ***P<0.001

## Taxonomy

### Tumoris

Huang, 2001: 98

http://species-id.net/wiki/Tumoris

Plate 1a, b, c, d

Absentia Huang, 2001: 58 (preoc., *Absentia* Togashi, 1990)
Absentia lanyuensis Huang - type species (=*Tumoris sanasaii* Huang, syn. n.)
Huangiella Kammerer, 2006: 269 (nom. nov. pro *Absentia* Huang )(=*Tumoris*, syn. n.)

#### Type species:

*Tumoris sanasaii* Huang, by original designation.

#### Redefinition of the genus.

Body spindle-shape, narrowing abruptly posteriorly; shield pentagonal, lobe present, with bulge between scapular tubercles, scapular tubercles set ahead of rear shield margin, seta directed upward; leg segments normal, coxae with 3 pairs of tubercles and seta, hind genual seta absent; empodium simple; opisthosoma differentiated into broader dorsal annuli and narrower ventral annuli, first dorsal annulus broad, fused forming a broad plate joined to prodorsal shield, dorsum with 3 ridges, median ridge ending before submedian ridges, the second ventral tubercle and setae (*e*) absent; coverflap with short ridges at base.

#### Differential diagnosis.

This genus is close to *Proneotegonotus* Mohanasundaram 1983, but differs from the latter by the absence of the second ventral tubercle and setae (*e*), presence of the first ventral tubercles and setae (*d*), and a bulge between the dorsal tubercles in prodorsal shield.

#### Classification.

In Huang (2001b) *Tumoris* was assigned to Tegonotini by the presence of lateral lobes in opisthosoma. After examining more specimens from several localities, we found the lateral lobes previously recognized were actually the submedian ridges on the dorsal opisthosoma (Pl. 1, b). According to the scapular tubercles located ahead of the rear shield, we re-assign this genus to Phyllocoptini.

**Plate 1.**
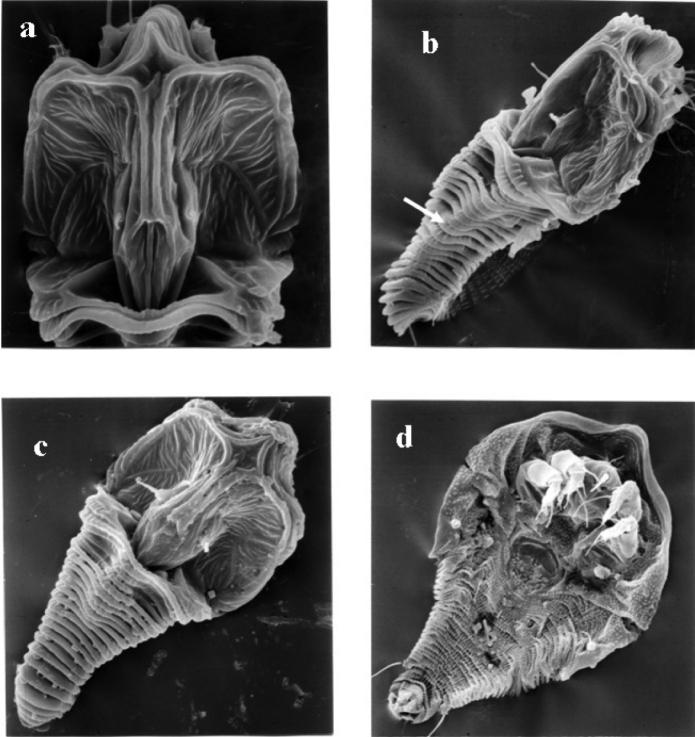
SEM micrographs of *Tumoris sanasaii* Huang, 2001. **a** prodorsal shield **b** lateral view, white arrow means submedian ridge **c** dorsal view **d** ventral view.

## Supplementary Material

XML Treatment for Tumoris
